# Correction: Rapid biocompatible macrocyclization of peptides with decafluoro-diphenylsulfone

**DOI:** 10.1039/c6sc90071b

**Published:** 2016-11-11

**Authors:** S. Kalhor-Monfared, M. R. Jafari, J. T. Patterson, P. I. Kitov, J. J. Dwyer, J. M. Nuss, R. Derda

**Affiliations:** a Department of Chemistry , University of Alberta , Edmonton , AB T6G 2G2 , Canada . Email: ratmir.derda@ualberta.ca; b Ferring Research Institute , San Diego , California 92121 , USA

## Abstract

Correction for ‘Rapid biocompatible macrocyclization of peptides with decafluoro-diphenylsulfone’ by S. Kalhor-Monfared *et al.*, *Chem. Sci.*, 2016, **7**, 3785–3790.



## 

In the original manuscript, an incorrect version of Fig. 1C in which the data points were inadvertently reflected around the vertical axis was shown. The corrected version of Fig. 1 appears below.
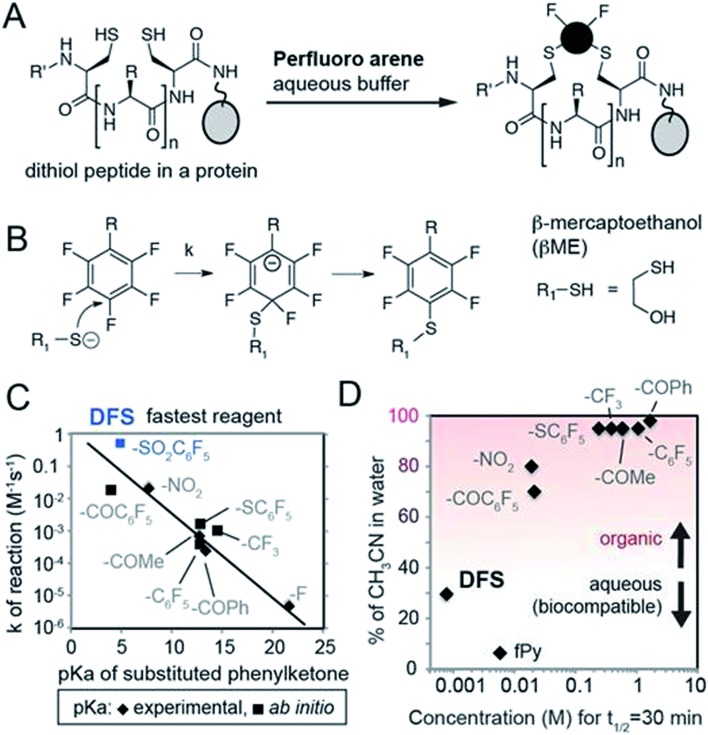


The Royal Society of Chemistry apologises for these errors and any consequent inconvenience to authors and readers.

